# Activation of propane on Ag–PdO(101) model surfaces

**DOI:** 10.55730/1300-0527.3757

**Published:** 2025-07-30

**Authors:** Mustafa KARATOK

**Affiliations:** Department of Nanotechnology and Nanomedicine, Graduate School of Natural Sciences, Hacettepe University, Ankara, Turkiye

**Keywords:** Heterogeneous catalysis, alkane oxidation, Ag-Pd alloys, bimetallic catalysts

## Abstract

Oxidation of alkanes remains a central challenge in catalysis due to the high activation barriers of C–H bonds and the thermodynamic favorability of complete oxidation. Palladium oxide (PdO), particularly its (101) facet, is known for its high reactivity in alkane oxidation, which is attributed to its coordinatively unsaturated palladium (Pd) and O atoms. In this study, we investigate the effect of silver (Ag) incorporation on the oxidation behavior of propane over PdO(101) using temperature-programmed reaction spectroscopy (TPRS) under controlled conditions. While pristine PdO(101) exhibits complete oxidation of propane with CO_2_ and H_2_O desorption at high temperatures (approximately 475 K), Ag incorporation induces a new CO_2_ desorption peak at significantly lower temperatures (approximately 330 K). This shift is attributed to the formation of new active sites at the Ag–PdO(101) interface. Quantitative analysis reveals that low-temperature activity correlates with Ag coverage, while overall CO_2_ production decreases, suggesting a redistribution of reactivity rather than an increase in active surface area. Activation energy estimations using the Redhead method confirm that C–H bond activation becomes more facile at the interface, with a 46 kJ/mol reduction compared to pristine PdO(101). These findings demonstrate that incorporating a less reactive metal such as Ag into PdO surfaces not only modifies the reaction energetics but also enables the design of bimetallic catalysts with improved selectivity for partial oxidation reactions.

## 1. Introduction

Oxidation reactions of saturated hydrocarbons, or alkanes, represent a vital class of chemical transformations with significant economic and environmental implications [1–3]. These reactions underpin practical applications such as power generation in gas turbines, where alkanes serve as fuel sources [4,5], and environmental remediation strategies, including the catalytic removal of unburned hydrocarbons from vehicle exhaust through complete oxidation to CO_2_ [6,7]. Beyond energy and emission control, alkane oxidation offers promising routes for producing value-added chemicals, such as alcohols, aldehydes, carboxylic acids, and other oxygenates [8–10]. Given the natural abundance and chemical inertness of alkanes, developing catalysts that can efficiently and selectively convert these molecules into partially oxidized products, rather than fully oxidized CO_2_, remains a central challenge in catalysis research. Success in this area would represent a major advancement toward more sustainable chemical manufacturing and energy utilization.

Alkanes exhibit weak interactions with most catalytic surfaces, making the initial C–H bond activation the rate-limiting step that involves a high energy barrier [11]. As a result, elevated temperatures are typically required to initiate catalytic transformations. However, operating under such high-temperature conditions, especially in the presence of oxidants, often leads to uncontrolled reaction pathways and favors the formation of CO_2_, the thermodynamically most stable product. This poses a major challenge in selectively producing partially oxidized compounds. Lowering the activation barrier for the first C–H bond cleavage would allow alkane activation under milder conditions, reducing the rate of complete oxidation and creating a window for controlling subsequent reaction steps and favoring the formation of valuable partial oxidation products. Achieving this level of control is a central objective in catalytic alkane conversion research.

Bimetallic nanoparticles have gained significant attention in heterogeneous catalysis due to their tunable and often enhanced chemical properties [12,13]. When two metals intermix and form an alloy, their individual electronic structures are modified, leading to substantial changes in how reactants bind to the catalytic surface [14]. As a result, bimetallic systems frequently exhibit improved catalytic activity, selectivity, and stability compared to their monometallic counterparts. In particular, alloys containing metals with inherently low reactivity toward organic molecules, such as gold (Au), silver (Ag), or copper (Cu), are often regarded as bifunctional catalysts [15]. In such systems, the more reactive metal typically facilitates the initial activation step, while the less reactive host metal modulates the reaction pathway to enhance selectivity toward the desired product. This synergistic behavior makes bimetallic nanoparticles especially promising for achieving selective oxidation of alkanes under controlled conditions.

While several transition metals and metal oxides have demonstrated high catalytic activity for alkane oxidation reactions, platinum-group metals, particularly Pt and Pd, exhibit exceptional performance under specific reaction conditions [16]. For instance, alumina-supported Pd catalysts are highly active in methane combustion under oxygen-rich conditions, which is the most challenging hydrocarbon oxidation reaction due to methane’s strong C–H bonds [17]. Despite the long-standing recognition of Pd’s high activity and numerous in-depth investigations [18,19], the nature of its active site remains under debate. Some studies have reported that PdO, rather than metallic Pd (Pd^0^), is responsible for alkane oxidation activity on Pd/alumina catalysts [20], while others argue that both Pd^0^ and PdO must coexist for optimal performance [21]. Alternatively, there are claims that partially oxidized Pd represents the most catalytically active state [22].

Experimental surface science studies employing well-defined single-crystal model surfaces have recently provided critical insights into this issue [23]. These investigations identified a specific oxide structure, namely PdO(101), as the active phase in alkane oxidation, in which coordinatively unsaturated Pd and O atoms serve as active sites for C–H bond activation in propane.

In this study, the reactivity of propane on Ag-decorated PdO(101) surfaces was investigated and compared with that on pristine PdO(101). Enhanced propane oxidation activity at lower temperatures was observed on Ag-decorated surfaces, which is attributed to new active sites formed at the Ag–PdO(101) interface. These findings offer direct evidence that incorporating an inherently less active metal such as Ag into Pd can facilitate C–H bond activation. This highlights the potential of bimetallic catalysts to enable selective oxidation and opens avenues for producing valuable partial oxidation products from alkanes.

## 2. Materials and methods

All experiments were performed in an ultrahigh vacuum (UHV) chamber maintained at a base pressure of 2 × 10^−10^ Torr. The system was equipped with a quadrupole mass spectrometer (QMS) (Hiden Analytical, Warrington, UK), an Auger electron spectrometer (AES) (model 15–155; Physical Electronics (PHI), Chanhassen, MN, USA), an electron gun for ion sputtering (model 161–5–251; Physical Electronics (PHI), Chanhassen, MN, USA), and an electron-beam evaporator (EFM3; Omicron Nanotechnology, Taunusstein, Germany). Prior to each measurement, the Pd(111) single crystal (10 mm diameter, 1.7 mm thickness, 6 N purity; Surface Preparation Laboratory, Zaandam, The Netherlands) was cleaned by repeated cycles of Ar^+^ ion sputtering and annealing. Argon gas (99.9999% purity; Matheson Tri-Gas, Basking Ridge, NJ, USA) was introduced at a background pressure of 1 × 10^−6^ Torr during sputtering, performed at 1 kV and 20 mA. The crystal was then annealed at 950 K for 5 min. After cleaning, the Pd(111) surface was oxidized by exposing it to controlled amounts of ozone at 500 K to generate the PdO(101) surface. Ozone was produced using a commercial electric discharge generator (LG-7; Ozone Engineering, Phoenix, AZ, USA) and dosed onto the sample via a leak valve. The ozone concentration in the oxygen stream was approximately 5%, monitored using a Teledyne 454H analyzer (Teledyne Instruments, Thousand Oaks, CA, USA), corresponding to about 60 g/N·m^3^.

Silver deposition onto the PdO(101) surface was carried out using the electron-beam evaporator, with a deposition rate maintained at 0.25 monolayers (ML) per minute. A silver rod (2.0 mm diameter, 99.99% purity; Surepure Chemicals Inc., Florham Park, NJ, USA) served as the evaporation source. The amount of deposited Ag was calibrated by Auger electron spectroscopy (AES) using a previously established calibration curve reported elsewhere [24]. For this calibration, Ag was deposited on clean Pd(111) surfaces at room temperature for varying durations at a constant deposition rate. A linear relationship between deposition time and Ag surface coverage was obtained [24] and was used in the present study to estimate Ag coverage on PdO(101). For temperature-programmed experiments, propane (99.99% purity; Matheson Tri-Gas, Basking Ridge, NJ, USA) was introduced onto the surface at 120 K using a leak valve. The sample was heated radiatively by a thoriated tungsten filament positioned behind the crystal. Sample temperature was measured with a K-type thermocouple inserted into a pinhole near the crystal’s edge. A proportional-integral-derivative (PID) controller (model 2404; Eurotherm Controls Inc., Ashburn, VA, USA) regulated the heating process, ensuring a constant rate of 1 K/s. Each surface, either PdO(101) or Ag/PdO(101), was freshly prepared prior to conducting a temperature-programmed experiment with propane.

## 3. Results and discussion

**Figure 1:**
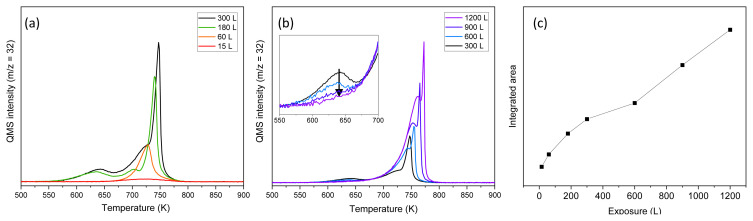
Temperature-programmed desorption spectra for oxygen (m/z = 32) following exposure of Pd(111) to ozone at 120 K: (a) ozone exposure from 15 to 300 L and (b) from 300 to 1200 L. All spectra were obtained at a constant heating rate of 1 K/s. (c) Integrated desorption areas corresponding to each ozone exposure, as shown in (a) and (b).

Formation of different oxide phases of Pd can be monitored through temperature-programmed desorption (TPD) of oxygen from the Pd(111) surface. The TPD profile changes with the total oxygen concentration accumulated on the surface, producing distinct desorption features. Upon exposure to ozone, which rapidly decomposes on reactive metal surfaces and deposits oxygen atoms, a series of O_2_ TPD spectra were collected following various ozone doses at 500 K (Figures 1a and 1b). At the lowest exposure level (15 Langmuir (L), 1L = 10^−6^ Torr·s), a broad desorption signal spanning from approximately 650 to 800 K was observed. Increasing ozone exposure to 60 L sharpened the signal around 730 K. These observations are consistent with prior studies, in which a broad desorption feature at low coverages was attributed to recombinative desorption of chemisorbed oxygen in a p(2 × 2) structure, while a sharper peak at higher coverages was linked to the decomposition of a two-dimensional (2D) Pd_5_O_4_ surface oxide layer [25].

Further increasing the ozone exposure to 180 L led to the development of three distinct desorption features at approximately 630, 700, and 730 K. These were assigned to the decomposition of PdO precursor states (PdO seeds), bulk-like PdO, and the surface oxide, respectively [26,27]. The PdO precursor state, composed of small oxide clusters formed upon oxidation of the 2D Pd_5_O_4_ layer, intensified with increasing oxygen coverage, as evidenced by the growing desorption signal at 630 K in Figure 1a. As these PdO seeds grow and transform into bulk-like PdO domains, the associated precursor desorption feature diminishes and eventually disappears, as highlighted in the inset of Figure 1b.

Upon further oxidation, exposure to 300 L of ozone resulted in the appearance of two desorption features centered at 720 and 750 K, indicative of bulk-like PdO decomposition (Figure 1b). With increasing ozone doses, these features shifted toward higher temperatures and grew in intensity. The emergence of an additional desorption peak at higher temperatures during bulk PdO decomposition has been explained by an autocatalytic mechanism [26]. In this scenario, oxygen atoms released from the decomposing bulk PdO migrate to the surface oxide layer, accelerating its desorption rate and producing a sharp high-temperature feature associated with surface oxide decomposition.

Oxygen desorption characteristics following ozone exposures above 300 L provide strong evidence for bulk-like PdO formation. As shown in Figure 1b, the disappearance of the precursor feature around 640 K, combined with the emergence of a new desorption peak at 720 K after 300 L of ozone exposure, indicates a transition to bulk oxide. The common leading edge of the 720 K signal suggests zero-order desorption kinetics, characteristic of desorption from a large reservoir of oxygen atoms and indicative of thick PdO layer formation. Additionally, a nonlinear relationship between oxygen uptake and ozone exposure further supports the sequential formation of distinct oxide phases. Figure 1c shows the integrated desorption areas as a function of ozone dose, revealing that the oxygen uptake rate initially declines up to about 600 L before transitioning to a linear increase. This change suggests that once a critical oxide thickness is reached, additional oxygen is incorporated into an oxide phase with consistent stoichiometry, consistent with the thickening of bulk-like PdO. These TPD findings are consistent with previous reports, reproducing characteristic features observed during PdO decomposition [27].

The structure of PdO formed through deep oxidation of Pd(111) is well established based on studies utilizing low-energy electron diffraction (LEED), X-ray photoelectron spectroscopy (XPS), low-energy ion scattering spectroscopy (LEISS), and computational methods [26–29]. This oxide predominantly exposes the (101) surface plane. In the bulk, each Pd atom is coordinated to four oxygen atoms; however, at the surface, two distinct types of Pd atoms exist based on their coordination numbers: fourfold (coordinatively saturated) and threefold (coordinatively unsaturated). These atoms are arranged in alternating parallel rows across the surface, resulting in half of the surface Pd atoms being coordinatively unsaturated. The high reactivity of the PdO(101) surface toward alkane activation has been attributed to the presence of these coordinatively unsaturated Pd sites [23,30].

**Figure 2:**
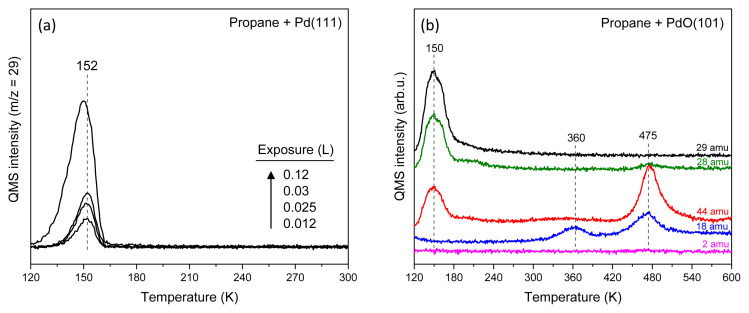
(a) Temperature-programmed desorption spectra showing molecular propane desorption from pristine Pd(111) following various propane exposures at 120 K. (b) Temperature-programmed reaction spectroscopy profiles illustrating the complete oxidation of propane on PdO(101). The heating rate was maintained at 1 K/s in all experiments.

Temperature-programmed reaction (TPR) experiments of propane on both Pd(111) and PdO(101) surfaces showed excellent agreement with previous studies [23]. Figure 2a presents TPR spectra showing various coverages of molecular propane desorption from a pristine Pd(111) surface after propane exposure at 120 K. In these measurements, the most intense cracking fragment of propane (m/z = 29) was monitored using a QMS. At low exposures, narrow desorption peaks centered at 152 K were observed, with peak intensity increasing as a function of propane exposure. Upon dosing 0.12 L (1 × 10^−9^ Torr for 120 s) of propane, a slight shift toward lower temperatures and noticeable peak broadening occurred. These features are attributed to the desorption of a physisorbed monolayer of propane on Pd(111), consistent with earlier reports [23]. The same study indicated that multilayer propane desorption occurs below 115 K, which is lower than the exposure temperature used in the present work; therefore, multilayer adsorption is not expected under these conditions.

Unlike pristine Pd(111), the PdO(101) surface activates propane and promotes its complete oxidation to CO_2_. Figure 2b presents TPR spectra for several mass fragments—m/z = 29 (propane), 28 (CO), 44 (CO_2_), 18 (H_2_O), and 2 (H_2_)—obtained after preparing a PdO(101) film by exposing Pd(111) to 900 L of ozone at 500 K, followed by propane dosing during cooling from 400 K down to 120 K, a process lasting over 15 min. To minimize site blocking by residual CO and H_2_ impurities in the chamber, propane (2 × 10^−9^ Torr) was introduced once the sample cooled to 400 K. This approach ensured the adsorption of propane at near-monolayer coverage on PdO(101), since multilayer accumulation typically occurs only at much lower temperatures [23]. Molecular propane desorption was observed near 150 K, as evidenced by the matching desorption features across the m/z = 29, 28, and 44 signals. A sharp m/z = 44 signal at approximately 475 K, accompanied by the absence of other propane fragments at this temperature, indicates that propane undergoes complete oxidation on PdO(101), forming CO_2_. The minor m/z = 28 signal detected at 475 K is attributed to the fragmentation pattern of CO_2_ in the mass spectrometer.

Further evidence for propane oxidation on PdO(101) is provided by the desorption of H_2_O, which must be produced in stoichiometric amounts during complete oxidation. Two distinct H_2_O desorption signals, centered at 360 and 475 K, were observed. These features are attributed to the oxidation of surface hydrogen atoms generated during the dissociation of the first and subsequent C–H bonds of propane, consistent with previous reports [23]. It has been proposed that below 200 K, propane undergoes initial C–H bond activation to form propyl species and hydrogen atoms, leading to a desorption-limited H_2_O signal at lower temperatures. The remaining propyl intermediates are subsequently oxidized at higher temperatures, giving rise to the reaction-limited H_2_O and CO_2_ signals above 400 K. Importantly, no H_2_ or partial oxidation products were detected, indicating that complete oxidation of propane is strongly favored on PdO(101). These observations are in excellent agreement with prior work [23].

**Figure 3:**
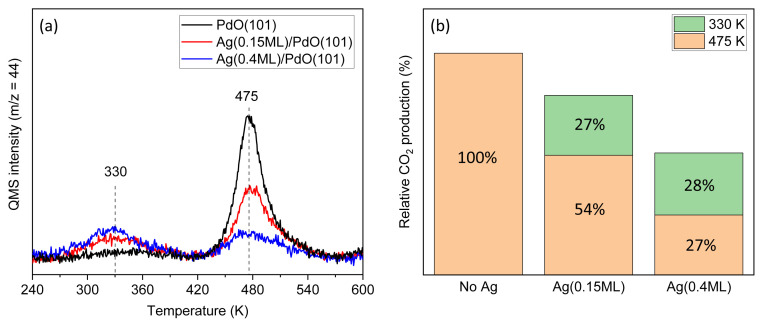
(a) Temperature-programmed desorption spectra showing CO_2_ desorption profiles from pristine and Ag-modified PdO(101) surfaces following propane exposure. A new CO_2_ desorption feature appears at approximately 330 K upon Ag incorporation, while the original peak at approximately 475 K diminishes with increasing Ag coverage. (b) Relative CO_2_ desorption areas at 330 K and 475 K, normalized to the total CO_2_ signal from pristine PdO(101), illustrating the redistribution of activity with increasing Ag coverage.

After establishing the complete oxidation behavior of propane on pristine PdO(101), the effect of silver incorporation on the reaction pathway was investigated. The incorporation of Ag into the PdO(101) surface led to a dramatic shift in the CO_2_ desorption temperature during propane oxidation (Figure 3a). Precalibrated amounts of Ag were deposited at 300 K onto the PdO(101) surface prepared as described above. The resulting Ag/PdO(101) surfaces were then exposed to propane during cooling to 120 K under a background pressure of 2 × 10^−9^ Torr. Temperature-programmed reaction profiles monitoring CO_2_ (m/z = 44) revealed the emergence of a new desorption peak at 330 K, whose intensity increased with Ag coverage (see Figure S1 in Supporting information for TPRS profiles). Concurrently, the original CO_2_ signal at 475 K diminished, clearly indicating that the low-temperature CO_2_ evolution is associated with Ag-modified sites. Notably, no oxygen desorption was observed during TPRS experiments below 600 K; however, the presence of Ag on PdO(101) altered the oxygen desorption characteristics, leading to a slight shift toward lower temperatures.

In contrast, the amount of CO_2_ produced at both 330 K and 475 K did not scale proportionally with the amount of Ag deposited. Relative CO_2_ production was quantified by integrating the desorption signals separately at 330 K and 475 K, as shown in Figure 3b. Compared to the pristine PdO(101) surface, CO_2_ production associated with the PdO(101)-related peak at 475 K decreased by 46% upon deposition of 0.15 ML of Ag, an amount corresponding to coverage of 15% of a clean Pd(111) surface area with a one-atom-thick layer, even though only a small fraction of the surface was modified with Ag. Meanwhile, the total CO_2_ production dropped by 19%. Increasing Ag coverage to 0.4 ML (corresponding to 40% surface coverage) further reduced the PdO(101)-related CO_2_ production to 27% of its original value, while total CO_2_ production decreased by 45%. These results, combined with the significantly higher oxygen affinity of Pd compared to Ag [31], strongly suggest that CO_2_ is not generated on Ag domains. Moreover, the comparable reductions in total CO_2_ production and corresponding Ag surface coverage (19% and 45% reductions for 15% and 40% Ag coverage, respectively) suggest that the low-temperature CO_2_ production originates from the Ag–PdO(101) interface. If Ag atoms or small clusters are well dispersed across the PdO(101) surface, they could block active Pd sites and influence the adsorption properties of neighboring Pd atoms. Such an effect would be consistent with the observed abrupt decrease in CO_2_ production at 475 K. Nevertheless, more detailed quantitative studies are necessary to confirm and further elucidate this hypothesis.

After observing a significant shift in the activation temperature for propane decomposition, the activation energies on the PdO(101) surface and at the Ag–PdO(101) interface were roughly estimated using the Redhead method [32], according to the following equation [33]:


$$E_{des}=RT_{max}\left[ln\left(\frac{\upsilon T_{max}}{\beta }\right)-3.46\right]$$

where E_des_ is the desorption activation energy, R is the gas constant, T_max_ is the temperature at peak desorption, υ is the preexponential factor, and β is the heating rate. Assuming first-order desorption kinetics, a constant preexponential factor of 10^15^ s^−1^ [23], and peak desorption temperatures of 330 K and 475 K, the activation energies were calculated to be 101 kJ/mol and 147 kJ/mol, respectively. Notably, the presence of Ag facilitates propane activation, lowering the activation energy by 46 kJ/mol compared to the pristine PdO(101) surface.

It should be noted that the Redhead method provides a simplified analysis that relies on several assumptions, and the activation energies calculated above may significantly deviate from the actual values. For example, although the CO_2_ desorption peak temperatures remain unchanged with varying Ag coverage, which is characteristic of first-order desorption, the actual propane coverage per Pd atom or per interfacial site may also remain constant, as the samples were exposed to excess propane. In such a case, the constant CO_2_ desorption temperatures would not necessarily reflect desorption kinetics associated with varying adsorbate coverages. Despite its limitations, the Redhead method offers a straightforward and practical approach for estimating activation energies from a single TPRS spectrum, allowing for approximate comparisons. Nevertheless, further experiments and a more rigorous kinetic analysis are required to clarify the role of Ag in modulating the reaction pathway.

It is also worth noting that no partial oxidation products were detected in any of the experiments described above. This outcome may be attributed to the presence of excess oxygen on PdO(101) domains, which readily react with intermediates and drive the reaction toward complete oxidation. Nonetheless, under different conditions, either on model systems or supported catalysts, Ag has the potential to promote selective oxidation, particularly if a weakly bound intermediate can spill over from the Ag–PdO interface onto Ag domains.

## 4. Conclusions

In this study, the activation and complete oxidation of propane were systematically investigated on PdO(101) and Ag-modified PdO(101) surfaces. Temperature-programmed desorption and reaction spectroscopy revealed that pristine PdO(101) is highly active for the complete oxidation of propane, producing CO_2_ and H_2_O as the sole products. The introduction of Ag onto PdO(101) led to a new low-temperature CO_2_ desorption feature centered at 330 K, suggesting the emergence of new active sites at the Ag–PdO(101) interface. Quantitative analysis indicated that the low-temperature CO_2_ production is associated with interfacial sites rather than Ag domains. Activation energy estimates further supported that propane activation becomes more facile in the presence of Ag, with a significant reduction of 46 kJ/mol. These findings highlight the critical role of metal–oxide interfaces in tuning hydrocarbon activation properties and provide valuable insights for the rational design of oxidation catalysts.

## Supporting information

Figure S1 Temperature-programmed reaction spectroscopy profiles showing complete oxidation of propane on Ag-decorated PdO(101) surfaces with Ag coverages of (a) 0.15 monolayers (ML) and (b) 0.40 ML. All experiments were conducted with a constant heating rate of 1 K/s.
