# Supplementary figures and images for: Activation of propane on Ag–PdO(101) model surfaces
Source: Turk J Chem. 2025 Jul 30;49(5):609–15. doi: 10.55730/1300-0527.3757 (PMC12604933; doi:10.55730/1300-0527.3757)

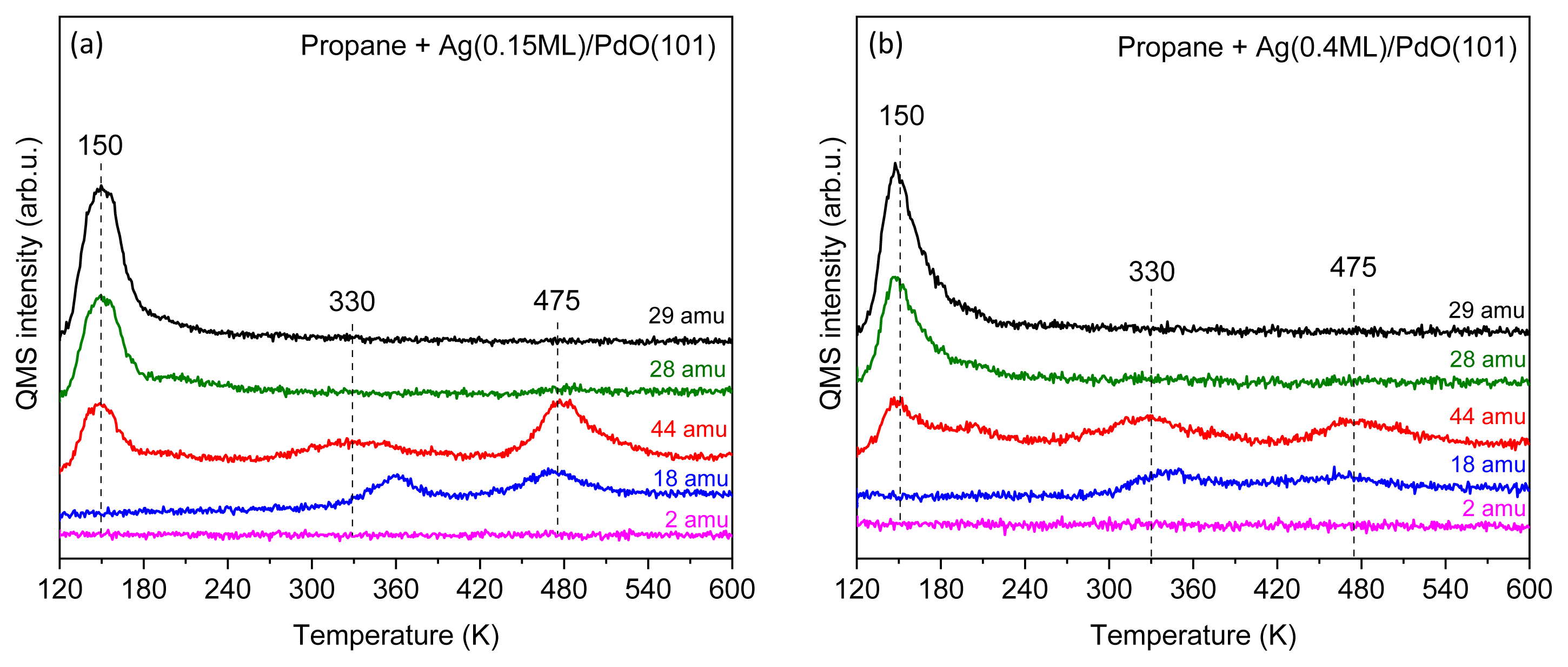

Supplement: Figure S1 — Temperature-programmed reaction spectroscopy profiles showing complete oxidation of propane on Ag-decorated PdO(101) surfaces with Ag coverages of (a) 0.15 monolayers (ML) and (b) 0.40 ML. All experiments were conducted with a constant heating rate of 1 K/s. [file tjc-49-05-609s1.tif]
